# Simultaneous Infection of *Elaphostrongylus* Nematode Species and Parasite Sharing between Sympatrically Occurring Cervids: Moose, Roe Deer, and Red Deer in Poland

**DOI:** 10.3390/pathogens10101344

**Published:** 2021-10-18

**Authors:** Magdalena Świsłocka, Mirosław Ratkiewicz, Anetta Borkowska

**Affiliations:** Department of Zoology and Genetics, Faculty of Biology, University of Białystok, Ciołkowskiego 1J, 15-245 Białystok, Poland; ermi@uwb.edu.pl (M.R.); abork@uwb.edu.pl (A.B.)

**Keywords:** *Alces alces*, *Capreolus capreolus*, *Cervus elaphus*, *Elaphostrongylus*, helminths, prevalence, nematode sharing

## Abstract

It is important to assess the distribution of parasite species across wildlife populations, to design strategies for game management and effective disease control in nature. In this project we quantified the prevalence of *Elaphostrongylus* species in eight moose populations. We used molecular methods for identification of parasite species and host individual genotypes from fecal samples. We also demonstrated sharing of parasite species between three cervid hosts sympatrically occurring in the Biebrza River valley, North-Eastern Poland, which is occupied by the largest autochthonous, non-harvested moose population in Central Europe. Nematode species from the genus *Elaphostrongylus* are ubiquitous in the studied moose populations. The presence of a single parasite species (e.g., *E. alces*) in moose individuals was more common than simultaneous infection with *E. alces* and *E. cervi*. The prevalence of both *E. alces* and *E. cervi* was higher in males than females. The distribution of *E. alces* and *E. cervi* prevalence in moose, roe deer, and red deer were in accordance with the membership of a host to a subfamily. Simultaneous occurrences of both *Elaphostrongylus* species were significantly more frequently noted in red deer fecal samples than those collected from moose or roe deer. Thus, we consider red deer to play a dominant role in sharing of those nematodes to other cervids. Our findings promote applications of molecular methods of identifying parasite species and the assessment of the exchange of parasite community between wild ruminant species in management and health monitoring of game animal populations.

## 1. Introduction

The assessment of the distribution of parasite species across wildlife populations is of basic and applied importance for the design of strategies for game management, restoration programs of large ungulate species, and an effective control of livestock diseases [1,2]. High population densities, human-driven changes to natural habitats and global climate, and an increasing distribution of alien species may favor the transmission of parasites between individuals [3,4]. Thus, wild animals often suffer from simultaneous infections with multiple parasite species [5]. The exchange of parasites between different host species is also common in nature, but most often requires sharing space such as pastures or waterholes, or intermediate hosts. Multihost pathogens pose a major problem for wildlife conservation, and their distribution in wild animal populations is still not well understood because past studies have focused most commonly on parasite sharing between wild and domesticated animals [6].

A great diversity of parasite species is noted in cervids [7]. Nematodes, and especially the cerebrospinal worms of the genus *Elaphostrongylus* (Protostrongylidae) are widely distributed in Eurasia [8]. *E. alces* mainly occurs in moose [9], but it can also infect reindeer, goats, and sheep [10]. As a generalist parasite, *E. cervi* is reported in different subspecies of red deer (*Cervus* spp.), but also in moose, roe deer, and domestic animals [9,11]. Adult parasites are located in the central nervous system and skeletal muscle fasciae, while eggs are transported with the blood to the lungs, embryonate there, and then L1 larvae leave the host with feces [12]. The ability of *Elaphostrongylus* spp. to infect ruminant species other than the usual host has been confirmed experimentally in several studies [13,14]. In natural conditions, the transmission between different cervids relies on ingesting infective stages of the parasites from gastropod intermediate hosts, due to the complex life cycle of all *Elaphostrongylus* species [8].

Cervidae are important game ungulates in Europe that share the same resources in most of their distribution ranges [2], thus the risk of cross-infection between them is high. Three native species of cervids are found in Poland: moose (*Alces alces*) and roe deer (*Capreolus capreolus*) from the Capreolinae subfamily (the New World deer), and red deer (*Cervus elaphus*) from the Cervinae subfamily (the Old World deer) [15]. The roe deer and red deer are the most numerous and widespread European ungulate species in Poland while moose reaches the western border of its range there. The distribution of moose in Poland is a result of spontaneous demographic expansion and successful reintroductions after the Second World War [16]. The hunting ban that was imposed in 2001 [17] is still in force across the country and enables the study of non-harvested moose populations that have currently reached high densities. The largest, genetically distinct population of moose occupies the Biebrza River valley [18], which is the most valuable natural complex of low bogs in Central Europe. The red deer was first reported in the Biebrza valley in the late 1970s [19]. Since then, its population has increased considerably, reaching over 1600 individuals. This area is also a habitat for over 3000 individuals of roe deer (combined official data from the Biebrza National Park, Rajgród, and Knyszyn State Forest, 2014).

The distribution of parasite species in moose populations in Poland, including the Biebrza valley, has been studied based on microscopic observation of eggs, oocysts, and larvae shedded in feces [20,21]. However, conventional methods did not allow to distinguish *Elaphostrongylus* species due to the overlapping morphological characteristics in some of the development stages. *E. alces* and *E. cervi* are clearly identified from non-invasive fecal samples by molecular assays [22]. DNA extracted from animal feces can be used not only for parasite gene amplification, but also for genetic identification of host individuals and their sex, which makes molecular techniques the ideal methods for parasite infection diagnosis in natural populations.

The aims of this present study are (1) to quantify the prevalence of *Elaphostrongylus* species in different moose populations using molecular methods for identification of both parasite species and host individual genotypes from non-invasively collected fecal samples, and (2) to demonstrate sharing of parasite species between sympatrically occurring cervid hosts.

## 2. Results

### 2.1. Occurrence of Elaphostrongylus Species in Moose Populations 

**Figure 1:**
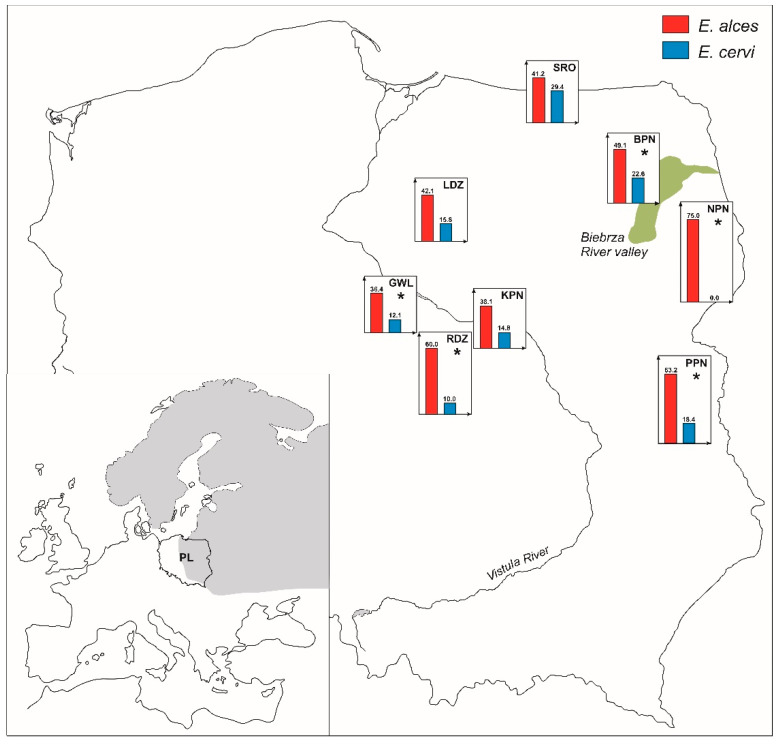
Prevalence (%) of *Elaphostrongylus alces* (in red) and *E*. *cervi* (in blue) in moose individuals from populations in Poland. Gray background on the map shows the distribution of moose in Europe. Population names: SRO—the Srokowo State Forest; LDZ—the Lidzbark State Forest; NPN— the Narwiański National Park; KPN—the Kampinos National Park; RDZ—the Radziwiłłów State Forest; GWL— the Gostynin-Włocławek Forests; PPN—the Polesie National Park. *—A significant difference in prevalence of parasite species in Chi-squared test. Data for BPN (the Biebrza National Park) population obtained from Świsłocka et al. [22].

The molecular analyses showed that endoparasites of both *Elaphostrongylus* species were present in seven out of the eight moose populations. *E alces* was noted in all moose populations with prevalence ranging from 36.4% (pop. GWL) to 75% (pop. NPN). No moose infected with *E*. *cervi* were found in the NPN population, whereas 29.4% of individuals from the SRO population were positive for this endoparasite species (Figure 1). The prevalence of *E*. *alces* in the whole sample (*N* = 217) was high and amounted to 50.2% (95% CI: 43.6–56.8), while for *E*. *cervi* it was 16.6% (95% CI: 12.2–22.1). In general, moose individuals were statistically more frequently infected with *E*. *alces* than *E*. *cervi* (*p* < 0.001) and the prevalence of *E*. *alces* was significantly higher than the prevalence of *E*. *cervi* in five out of the eight moose populations: NPN, RDZ, GWL, PPN, and BPN (*p* < 0.05; Figure 1). 

*E. alces* endoparasite was detected in fecal samples of both sexes of moose, and the positive rates in males and females ranged in the studied populations from 33.3–66.7% and 20.3–85.7%, respectively (Table 1). *E. cervi* did not occur in males from the NPN and KPN populations and females from the NPN, RDZ, and GWL populations (Table 2). Significant differences in prevalence of both nematode species were observed between sexes in the whole sample and in the GWL population, with higher prevalence in males than females (Table 1 and Table 2).

Overall, no DNA of brainworms was detected in 43.4% of moose fecal samples. Infections with *Elaphostrongylus* nematodes were significantly more prevalent as 56.7% (123 out of 217, CI: 50.0–63.1) of moose were positive for at least one *Elaphostrongylus* species (χ^2^ = 7.75; *p* < 0.001). Infections with a single parasite species were significantly more common (46.5%) than simultaneous infections with two parasite species (10.1%; χ^2^ = 4.46; *p* < 0.05). In particular, one parasite species (*E. alces* or *E. cervi*) was more frequently found than two species (*E. alces* and *E. cervi*) in the SRO, NPN, RDZ, PPN, and BPN populations (*p* < 0.05; Table 3).

### 2.2. Simultaneous Infection with Elaphostrongylus Species in Sympatrically Occurring Cervids

**Figure 2:**
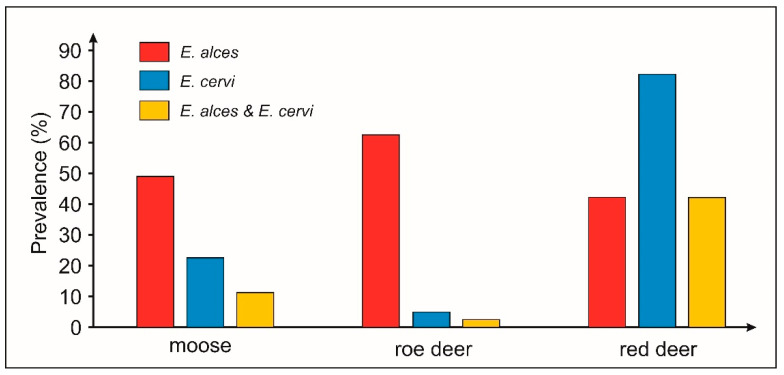
Infection rate (prevalence) with *Elaphostrongylus* species: *E*. *alces* (in red), *E*. *cervi* (in blue) and co-infection with two parasite species (*E. alces* and *E. cervi*; in yellow) in the moose *Alces alces*, the roe deer *Capreolus capreolus* and the red deer *Cervus elaphus* from the Biebrza valley, NE Poland.

Molecular analyses showed that 26 individual moose (49.1%), 25 roe deer (62.5%), and 19 red deer samples (42.2%) from the Biebrza River valley were positive for *E*. *alces* parasite (Figure 2). The moose and the roe deer were significantly more frequently infected with this nematode species than with *E*. *cervi* (*p* < 0.001). In red deer, the prevalence of *E. cervi* was at 82.2%, which is significantly higher than in moose (22.4%) and roe deer (5%), and the infection rate with *E. alces* at 42.2% (Figure 2). Simultaneous occurrences of both *Elaphostrongylus* species were also more frequently noted in the red deer fecal samples (19/45) than those collected from moose (6/53; χ^2^ = 14.36; *p* < 0.002) and the roe deer (1/40; χ^2^ = 18.57; *p* < 0.001). 

## 3. Discussion

The study provides clear evidence that nematodes from the genus *Elaphostrongylus* are ubiquitous moose parasites in Poland. The presence of a single parasite species (e.g., *E. alces*) in moose individuals is more common than simultaneous infections with *E. alces* and *E. cervi*. The Eurasian moose could be a host for a great number of parasite species [23,24], and parasitic infections in these animals almost always occur in an extremely high intensity and prevalence, unusual for other species of wild ruminants [24]. *E. alces* nematode can cause a neurologic disease called elaphostrongylosis, resulting in death of moose calves and yearlings, which was reported in Sweden in the 1980s [25]. The epizootiology of *E. alces* in Swedish moose was broadly investigated based on animal dissections [26]. In Poland, the first records of adult specimens of *E. alces* in moose tissue and its larvae in fecal samples were made in 2005 in Kampinos National Park [27]. In the present study, *E*. *alces* was found in all moose populations and between 36% and 75% of moose individuals were infected by this parasite species. Interestingly, the lowest prevalence of this endoparasite was noticed in the GWL population, localized at the western margin of the moose range in Poland, while the eastern populations (NPN and PPN) were characterized by the highest parasite frequencies. In Sweden, the parasite was distributed throughout the country in the 1980s, with the highest prevalence (56%) in the central region and lowest (13%) in the south. Calves and adults over 9 years of age were more often infected than middle-aged animals [26]. The percentage of old individuals in the non-harvested Polish populations is high, likely due to the hunting ban on moose over the last 20 years, which could explain the high values of *E*. *alces* prevalence. 

As expected, the overall prevalence of both *E. alces* and *E. cervi* differed between sexes in moose, with higher prevalence in males than females. However, significant sex-biased differences were observed only in the GWL population. Among vertebrates, prevalence and intensity of parasitism is often higher in males than females. It is mainly caused by the effect of sex hormones on immunity and differential exposure to parasites [28,29,30]. Results of studies concerning the sex-biased occurrence of *Elaphostrongylus* spp. that have been carried out so far are ambiguous. Stuve [31] showed higher prevalence of *E. cervi* among males than females in moose from Norway, based on the observation of eggs and larvae in feces. The red deer males also presented higher *E. cervi* L1 counts than females [32]. However, Steen et al. [26] did not note any statistical differences between sexes, but they found that the prevalence of *E. alces* trended higher in young males during the examination of moose tissues. The prepatent period varies among the *Elaphostrongylus* spp. and infected host species [11]. For *E. alces*, the prepatent period is between 39 and 73 days in moose and up to 4 months in abnormal hosts [33]. Additionally, the excretion of *Elaphostrongylus* sp. larvae in moose feces varied between seasons, peaking in February [21]. Some individuals, which proved to be negative for parasite DNA in their fecal samples, might nevertheless have been infected. The differences in the time at which fecal samples were obtained may additionally alter the estimation of parasite occurrence in a population. Many endoparasite species, such as nematodes, cannot be identified with certainty using conventional methods, because the development stage, such as eggs and larvae, which are more easily obtainable from fecal samples, are less distinguishable especially at the species level [34]. Our study confirmed that molecular methods are precise and highly effective, with great potential for diagnosis of parasitosis in wild cervids. We were able to determine the occurrence of two different species of *Elaphostrongylus* which were often identified at genus level based on morphological characteristic of eggs and/or larvae [21,35]. This limitation of conventional methods could be one of the main reasons for underestimating the real dispersion of different brainworm species in moose populations and the level of cross-transmission between different host species. 

Parasites of the genus *Elaphostrongylus* are capable of maturing and producing larvae in other animal species than the final host with which they are phylogenetically associated [13,14,33]. *E. alces* is regarded to be evolutionarily adapted to moose [26] while *E. cervi* is classified as a generalist among parasites that infect variable hosts [1]. This study suggested that all members of subfamily Capreolinae should be considered natural hosts for *E. alces*, as both moose and roe deer were infected with this nematode species with the high prevalence of 49–62% and significantly more frequently than with *E*. *cervi*. On the other hand, the typical moose nematode species, *E*. *alces* occurred in 42% of the red deer fecal samples from the Biebrza valley, indicating its invasive character and a well-established host/parasite relationship in the Cervinae subfamily. On the other hand, the prevalence of *E. cervi* was significantly higher in the red deer (82%) than in moose (22%) and roe deer (5%). Stuve [31] considered moose to be a natural host for *E. cervi*, since this parasite species was present in the moose population throughout southern Norway with the infection rate of 19–43%. In Poland, the level of *E. cervi* parasitism was lower and varied between moose populations and varied between moose populations from 10 to 29%. *E. cervi* was not present only in the population characterized by the highest prevalence of *E. alces* (NPN; Figure 1). 

Host-specific parasites constitute fewer than half of the total nematode species in wild and domestic ungulates [6]. Host sharing is variable but prevalent between closely related host species [1]. The transmission of pathogens between different hosts in the natural environment occurs indirectly, through shared space and vectors, rather than via direct interspecies contact. Deer infect themselves with *Elaphostrongylus* nematodes by consuming them with grass or plant shoots and small terrestrial snails infected with invasive larvae [36]. For nematodes with a complex life cycle density dependent parasitism is not expected. No relationship was found between the prevalence of *E. cervi* and the population density of moose [31], and red deer [37]. Theoretically, as host population density increases, individuals are more likely to encounter infective parasite stages. Thus, the risk of both direct and indirect parasite cross-transmission could be greater in areas of high densities of the host species which could potentially exchange parasites. Sharing space and resources by closely related host species will provide additional opportunities for host-switching. Based on molecular analysis in this study, *E. cervi* was detected in a majority of moose population in Poland where red deer is numerous and widespread [38]. *E. cervi* also infected over 20% of moose in the Biebrza valley [22] where red deer has been reported for the last fifty years [19]. These results correspond to the earlier study from Norway, in which the highest prevalence of *E. cervi* infection in moose was found where red deer also occurred and harbored *E. cervi* [31]. 

In our molecular analysis, simultaneous occurrences of both *Elaphostrongylus* species were more frequently noted in red deer fecal samples than those collected from moose or roe deer. Infections by different and usually numerous parasite species are frequently observed at individual, population and host species levels [5]. Widespread heterogeneities in parasite loads suggest that some host individuals or species with a higher susceptibility or infectiousness may act as super-spreaders and disproportionately contribute to parasite transmission [39]. For example, Escobar et al. [40] found the high transmission risk of a protostrongylid nematode *Parelaphostrongylus tenuis* to areas inhabited by moose populations with a high density of white-tailed deer (*Odocoileus virginianus*). From this perspective, red deer could be a reservoir of both species of genus *Elaphostrongylus* and play a dominant role in transmission of these cerebrospinal nematodes to other cervid populations in the Biebrza valley and all over the country. This is of particular importance in the context of the spectacular expansion of deer species observed in Europe during the last 3–5 decades, which is a result of successful restoration programs and conservation science [41].

## 4. Materials and Methods

### 4.1. Data Collection and Genetic Analyses

Fresh fecal samples of three cervid species were collected from November to March between 2008 and 2012 in eight moose populations from NE Poland (Figure 1), and between 2012 and 2013 in roe deer, and red deer populations localized in the Biebrza River valley. Stool samples were cooled and frozen at −20 °C, at which point molecular analyses were possible. DNA extraction and purification from ~0.025 g of fecal samples were performed using the QIAamp DNA Stool Mini Kit (Qiagen, Hilden, Germany), according to the manufacturer’s protocol. In the next step, to avoid pseudo-replication, DNA from each moose fecal sample was examined for 11 microsatellite loci and ~333-bp fragment of the SRY gene to molecularly identify individuals and their sex [18,42]. The 201 multi-locus microsatellite profiles were assigned to 164 different moose individuals (68 males and 96 females) using CERVUS 3.0.3 [43] from the Srokowo State Forest (pop. SRO; *N* = 17), the Lidzbark State Forest (pop. LDZ; *N* = 19), the Narwiański National Park (pop. NPN; *N* = 16), the Kampinos National Park (pop. KPN; *N* = 21), the Radziwiłłów State Forest (pop. RDZ; *N* = 20), the Gostynin-Włocławek Forests (pop. GWL; *N* = 33), and the Polesie National Park (PPN; *N* = 38). Genetic analyses were not carried out to identify different individuals and their gender among roe deer (*N* = 40) and red deer (*N* = 45) fecal samples collected from the Biebrza River valley. To avoid multiple sampling from the same individual of roe deer and red deer, their fecal samples were collected over several days from different localizations in the Biebrza valley, several dozen kilometers away from each other.

The DNA isolated from the fecal samples of the three host species were screened for infection with two cerebrospinal nematodes: *Elaphostrongylus alces* and *E. cervi* (Protostrongylidae). Fragments of Internal Transcribed Spacer 2 (ITS2) coding regions were amplified using primers designed for *E*. *alces* and *E*. *cervi* by Świsłocka et al. [22]. PCR reactions were performed with ~25 ng genomic DNA, 1.7 μL Qiagen multiplex PCR Master Mix (1×), 0.3 μL mix of species-specific primers, and 1 μL Qiagen RNase-free water in a 5 μL reaction volume (Qiagen, Germany). The PCR amplification of the ITS2 fragments was performed in a Labcycler Gradient (SensoQuest, Goettingen, Germany) with the following profile: initial denaturation at 95 °C for 15 min, followed by 35 cycles consisting of denaturation at 94 °C for 30 s, annealing at 57 °C for 90 s, extension at 72 °C for 60 s, and final extension step at 60 °C for 30 min. 

PCR products were identified by electrophoresis on 1.5% agarose gel. The specific DNA fragments of the ITS2 gene: 200-bp for *E*. *alces* and 156-bp-long for *E*. *cervi* were confirmed after GelRed (GeneON, Ludwigshafen, Germany) staining under UV light using a transilluminator. To evaluate the correctness of the agarose gel electrophoretic method in distinguishing *Elaphostrongylus* species, eight PCR-positive samples for *E*. *alces* and eight samples for *E*. *cervi* were sequenced. For this purpose, the chosen PCR-positive samples were purified with shrimp alkaline phosphatase (SAP) and Exonuclease I (Thermo Scientific, EU Thermo Fisher Scientific, Waltham, MA, USA) in an enzymatic reaction following the manufacturer’s instructions. Then, they were processed for cycle sequencing PCR with a BigDye^TM^ Terminator v3.1 Cycle Sequencing Kit v.3.1 (Applied Biosystems, Foster City, CA, USA). Unincorporated dideoxynucleotides were eliminated from the sequencing reaction using the ExTerminator Kit (A&A Biotechnology, Gdansk, Poland). The sequencing products were run on an automated capillary sequencer ABI 3130 (Applied Biosystems, Foster City, CA, USA). The resulting sequences were edited and aligned in BioEdit v.7.0.4 [44], revised manually, and compared to the GenBank references (AF504034 for *E*. *alces* [45], KY081650 for *E*. *cervi* [46]) by BLAST (http://www.ncbi.nlm.nih.gov/, accessed on 15 september 2021) to determine the parasite species.

### 4.2. Statistical Analyses

The proportion of infected individuals/samples (prevalence) within the moose, the roe deer and the red deer populations and their 95% confidence intervals (CI) were calculated using OpenEpi (http://www.openepi.com/Proportion/Proportion.htm, accessed on 15 September 2021) based on Wilson score interval. The prevalence of *E*. *alces* and *E*. *cervi* parasites in the moose population from the Biebrza valley (pop. BPN; *N* = 53) were obtained from Świsłocka et al. [22]. Additionally, the prevalence of both endoparasite species was determined separately for males and females of moose. The number of parasite species per host (parasite species richness) was calculated for the three studied cervids from the Biebrza valley. Statistical analysis was performed using the Chi-squared test in OpenEpi (http://openepi.com/TwobyTwo/TwobyTwo.htm, accessed on 15 September 2021). We tested for statistically significant differences in the prevalence of two *Elaphostrongylus* species between the moose populations and sexes and between three deer species: moose, roe deer, and red deer sympatrically occurring in the Biebrza River valley. Statistical differences in the frequency of infection of one or two *Elaphostrongylus* species in different host populations from the Biebrza valley were also evaluated. *P* values smaller than 0.05 were considered to indicate significant variation.

## 5. Conclusions

Nematode species from the genus *Elaphostrongylus* are ubiquitous in the studied moose populations. Molecular analyses made it possible to assign individual genotypes and sexes to hosts, and to distinguish parasite species from non-invasively collected fecal samples. As expected, the overall prevalence of both *E. alces* and *E. cervi* differed between sexes, with higher prevalence in males than females. The distribution of *E. alces* and *E. cervi* prevalence in moose, roe deer and red deer was in accordance with the phylogenetic pattern and the host’s subfamily. However, *E. alces* infections, frequently noted in red deer, point to the invasive character of this parasite and a well-established host/parasite relationship also in the Cervinae subfamily. We consider red deer to be a reservoir of both species of genus *Elaphostrongylus* which plays a dominant role in the transmission of these cerebrospinal nematodes to other sympatrically occurring cervids. The assessment of the exchange of parasite communities between wild ruminant species is of particular importance for monitoring health and managing moose and other game mammal populations in Europe and should be the focus of further comprehensive molecular-based studies.

## Figures and Tables

**Table 1:** Prevalence of *Elaphostrongylus alces* obtained from molecular analysis of moose individual fecal samples from eight populations in Poland.

Population	No. Males	Males		No. Females	Females		*p* Value
		No. Positive	% (CI)		No. Positive	% (CI)	
SRO	7	4	57.1 (25.0–60.3)	10	3	30.0 (10.8–60.3)	0.264
LDZ	8	5	62.5 (30.6–86.3)	11	3	27.3 (9.8–56.6)	0.125
NPN	9	6	66.7 (35.4–87.9)	7	6	85.7 (48.7–97.4)	0.383
KPN	3	1	33.3 (6.2–79.2)	18	7	38.9 (20.3–61.4)	0.854
RDZ	13	8	61.5 (35.5–82.3)	7	4	57.2 (25.0– 84.2)	0.848
GWL	13	8	61.5 (35.5–82.3)	20	4	20.0 (8.1–41.6)	0.015
PPN	15	9	60.0 (35.8–80.2)	23	15	65.2 (44.9–81.2)	0.745
BPN *	26	16	61.5 (42.5–77.6)	27	10	37.04 (21.5–55.8)	0.075
All	94	57	60.6 (50.5–69.9)	123	42	34.1 (26.4–42.9)	0.0001

* *p* < 0.05.

**Table 2:** Prevalence of *Elaphostrongylus cervi* obtained from molecular analysis of moose individual fecal samples from eight populations in Poland.

Population	No. Males	Males		No. Females	Females		*p* Value
		No. Positive	% (CI)		No. Positive	% (CI)	
SRO	7	3	42.9 (15.8–75.0)	10	2	20.0 (5.7–51.0)	0.311
LDZ	8	2	25.0 (7.2–59.2)	11	1	9.1 (1.6–37.7)	0.348
NPN	9	0	0	7	0	0	−
KPN	3	0	0	18	3	16.7 (5.8–39.2)	0.459
RDZ	13	2	15.4 (4.3–42.2)	7	0	0	0.275
GWL	13	4	30.8 (12.7–57.6)	20	0	0	0.008
PPN	15	3	20.0 (7.0–45.2)	23	4	17.4 (7.0–37.1)	0.839
BPN *	26	7	26.9 (13.7–46.1)	27	5	18.5 (8.2–36.7)	0.465
All	94	21	22.3 (15.1–31.8)	123	15	12.2 (7.5–19.2)	0.046

* *p* < 0.05.

**Table 3:** Fecal samples of moose individuals with no *Elaphostrongylus* sp., positive for one (*E. alces* or *E*. *cervi*) and two parasite species (*E. alces* and *E. cervi*).

Pop.	No. Sample	No Parasite Species		One Parasite Species		Two Parasite Species	
		No. Negative	% (CI)	No. Positive	% (CI)	No. Positive	% (CI)
SRO	17	7	41.2 (21.6–64.0)	8	47.0 (26.2–69.0)	2	11.8 (3.3–34.3)
LDZ	19	11	57.9 (36.3–76.9)	5	26.3 (11.8–48.8)	3	15.8 (5.5–37.6)
NPN	16	4	25.0 (10.2–49.5)	12	75.0 (50.5–89.8)	0	0
KPN	21	12	57.2 (36.6–75.5)	7	33.3 (17.2–54.6)	2	9.5 (2.7–28.9)
RDZ	20	7	35.0 (18.1–56.7)	12	60.0 (38.7–78.1)	1	5.0 (0.9–28.9)
GWL	33	21	63.6 (46.6–77.8)	8	24.3 (12.8–41.0)	4	12.1 (4.8–27.3)
PPN	38	11	29.0 (17.0–44.8)	23	60.5 (44.7–74.4)	4	10.5 (4.2–24.1)
BPN *	53	21	39.6 (27.6–53.1)	26	49.1 (36.1–62.1)	6	11.3 (5.3–22.6)
All	217	94	43.4 (36.9–50.0)	101	46.5 (40.0–53.2)	22	10.1 (6.8–14.9)

* *p* < 0.05.

## Data Availability

The data that support the findings of the study are available from the corresponding author.
